# COVID-19 and risk management in a tissue bank

**DOI:** 10.6061/clinics/2020/e2132

**Published:** 2020-07-06

**Authors:** André Oliveira Paggiaro, Renata Oliveira Conceição, Mariana Parra Bianchi, Rolf Gemperli

**Affiliations:** Departamento de Cirurgia Plastica e Banco de Tecidos, Hospital das Clinicas HCFMUSP, Faculdade de Medicina, Universidade de Sao Paulo, Sao Paulo, SP, BR

Tissue banks (TBs) are establishments that have the physical infrastructure, equipment, and human resources necessary for competently screening, withdrawing, identifying, transporting, evaluating, processing, packaging, storing, and distributing human tissue for therapeutic use (1). To evaluate the methodologies of the various processes performed in a TB, Quality Management Systems (QMSs) are set up to address aspects such as personnel training, quality control, corrective actions, guaranteed traceability, and accurate documentation, among others. The fundamental objective of the QMS is to reduce the risk of disease transmission to tissue recipients (2).

Recently, the coronavirus disease (COVID-19) pandemic has evoked a new challenge for TB QMSs. This is primarily because little is known about the possibility of human tissue contamination or the risk of disease transmission following transplantation. Although, until now, there have been no documented reports of severe acute respiratory syndrome coronavirus 2 (SARS-CoV-2) transmission following tissue transplantation, the Instituto Central do Hospital das Clínicas (ICHC) TB aimed at establishing preventive actions to reduce the possibility of allograft contamination.

In early March 2020, the first few cases of community transmission of COVID-19 in the state of São Paulo were identified. Even though TBs are an essential health service, given the current uncertainties, we opted to suspend the collection of new skin and amniotic membrane samples until better security measures were established to manage the situation.

On March 25, 2020, the Ministry of Health issued a memorandum regulating the technical criteria for the definition of organ and tissue donors during the pandemic (3). The memorandum was centered around the delimitation of strict clinical criteria for donor selection, the use of reverse transcription-polymerase chain reaction (RT-PCR) for screening donors, and the determination of protective measures for the health professionals involved. As regards skin transplantations, the standard stipulates that tissue collection must be performed with caution in regions of community transmission, thus transferring the responsibility for establishing strict safety protocols to the TBs.

In response to the memorandum, the ICHC TB QMS started developing strategies to cope with the crisis, focusing on three fundamental aspects: care in donor selection and tissue processing, safety of personnel, and quality assurance of previously stored tissue stock.

## CARE IN DONOR SELECTION AND TISSUE PROCESSING

Analysis of an individual’s medical history is essential when selecting tissue donors. In this regard, during the commencement stage of SARS-CoV-2 community transmission, we included symptoms of suspected COVID-19 such as severe acute respiratory syndrome and/or previous contact with confirmed cases as criteria for refusal. Notably, the Central de Transplantes Regional de São Paulo began collecting nasal swabs and testing every organ donor and tissue donor for COVID-19 using RT-PCR.

Taken together, obtaining clinical history and RT-PCR results ensured greater security for the TB QMS, reduced the potential risk of allograft contamination to a level that we felt was safe, and led to the decision to resume skin sample collection. However, lack of funding from Sistema Único de Saúde for the processing of amniotic membrane samples made it impossible for the ICHC TB to perform RT-PCR examinations in all pregnant women, and, as a consequence, we have not been able to reinitiate the collection of this tissue.

The expression and distribution of hACE2 receptors in different human cells can be used to identify potential routes of entry of the virus, and these receptors have been shown to be present on keratinocytes (4). Thus, the skin is a potential target for SARS-CoV-2 infection. This possibility is further supported by a recent study that reported the presence of skin manifestations such as rash, urticaria, and vesicles in 18 patients with COVID-19 (5). However, it is noteworthy that there are currently no studies that have evaluated the effects of allograft processing on the viral load of the new coronavirus.

At the ICHC TB, we use glycerolation to preserve tissue, and glycerol has been shown to possess decontaminating and virucidal actions (6). While it is plausible that glycerolation could effectively inactivate Coronaviridae-type viruses, there have been no studies that validate this possibility. We also use ionizing irradiation as a complementary sterilization method when glycerolated skin is contaminated by Gram-positive bacteria and fungi. Previous laboratory tests have demonstrated that ionizing irradiation is effective in inactivating other coronaviruses (SARS-CoV and MERS-CoV) (7)(8), but its effect on SARS-CoV-2 is still unknown. On the basis of these facts, we decided to subject all newly collected tissues to complementary sterilization with a 25 kGy dose of radiation. It is believed that this added layer of security will increase the quality and safety of the transplanted tissue.

## SAFETY OF PERSONNEL

Even under normal circumstances, special care is taken at TBs to guarantee that the tissues are not contaminated and the personnel are safe − for example, through the use of clean rooms, laminar flow hoods, properly sterilized material, and high-quality surgical dressing and ensuring compliance with good laboratory practice guidelines. While all professionals working at the ICHC TB have received excellent safety training, they have recently undergone reorientation regarding the importance of using personal protective equipment, currently wear N95 masks during tissue collection and processing, and are frequently reminded of the importance of wearing goggles. Recommendations about hand hygiene and avoiding unnecessary crowds and physical contact have also been made. Furthermore, employees working in administrative areas have also been provided surgical masks, which they are required to wear.

## QUALITY ASSURANCE OF PREVIOUSLY STORED TISSUE STOCKS

Another important point for consideration concerning the QMS is defining the exact moment that viral circulation began in a given region. While the first confirmed case in Brazil was recorded on February 26, 2020, the possibility of undetected viral circulation before this date cannot be excluded. A notable example of such a situation comes from a recent Italian study that showed that the virus was circulating throughout Italy for weeks before the first case was identified (9).

The ICHC TB collected tissues at the beginning of the year, and potential risk of contamination of these allografts cannot be ruled out. We have, therefore, reassessed all of the medical records to identify any signs in the clinical history that could be indicative of SARS-CoV-2 infection at the time of tissue collection, and, in some cases, we have contacted the family members of the donors for further clarification. Additionally, we have elected to irradiate all stored lots collected in 2020 with a 25 kGy radiation dose.

## CONCLUSION

Following establishment of the contingency protocol in response to the COVID-19 pandemic and the implementation of corrective measures and training of personnel, the ICHC TB resumed its normal activities on April 24, 2020. Since then, three new skin collections have been made, totaling 6500 cm^2^ of tissue, which is sufficient for treating three to four patients with extensive burns. Additionally, three patients have undergone skin allograft transplantations, using a total of 7060 cm^2^ of skin, and no adverse events have been reported as yet. Furthermore, all professionals at the ICHC TB have been tested for SARS-CoV-2, and, so far, none have tested positive. Taken together, we can confidently state that the ICHC TB was able to develop an effective plan for maintaining the health service with high levels of quality assurance even during a pandemic.
